# Supplementary material for: Genomic and transcriptomic variation defines the chromosome-scale assembly of Haemonchus contortus, a model gastrointestinal worm
Source: Commun Biol. 2020 Nov 9;3:656. doi: 10.1038/s42003-020-01377-3 (PMC7652881; doi:10.1038/s42003-020-01377-3)
Supplement: Supplementary file 1 — Supplementary Information [file 42003_2020_1377_MOESM1_ESM.docx]

Supplementary Information

**Genomic and transcriptomic variation defines the chromosome-scale assembly of *Haemonchus contortus*, a model gastrointestinal worm**

Stephen R. Doyle^1*^, Alan Tracey^1^, Roz Laing^2^, Nancy Holroyd^1^, David Bartley^3^, Wojtek Bazant^1^, Helen Beasley^1^, Robin Beech^4^, Collette Britton^2^, Karen Brooks^1^, Umer Chaudhry^5^, Kirsty Maitland^2^, Axel Martinelli^1^, Jennifer D. Noonan^4^, Michael Paulini^6^, Michael A. Quail^1^, Elizabeth Redman^7^, Faye H. Rodgers^1^, Guillaume Sallé^8^, Muhammad Zubair Shabbir^9^, Geetha Sankaranarayanan^1^, Janneke Wit^7^, Kevin L. Howe^6^, Neil Sargison^5^, Eileen Devaney^2^, Matthew Berriman^1^, John S. Gilleard^7^, James A. Cotton^1*^

1. Wellcome Sanger Institute, Hinxton, Cambridgeshire, CB10 1SA, United Kingdom
2. Institute of Biodiversity Animal Health and Comparative Medicine, College of Medical, Veterinary and Life Sciences, University of Glasgow, Garscube Campus, Glasgow, G61 1QH, United Kingdom
3. Moredun Research Institute, Pentlands Science Park, Bush Loan, Penicuik EH26 0PZ, United Kingdom
4. Institute of Parasitology, McGill University, 21111 Lakeshore Road, Sainte Anne-de-Bellevue, Québec, H9X3V9 Canada
5. Royal (Dick) School of Veterinary Studies, University of Edinburgh, Edinburgh, EH25 9RG, United Kingdom
6. European Molecular Biology Laboratory, European Bioinformatics Institute, Hinxton, Cambridgeshire, CB10 1SA, United Kingdom
7. Department of Comparative Biology and Experimental Medicine, Faculty of Veterinary Medicine, University of Calgary, Calgary, Alberta, Canada
8. INRAE - U. Tours, UMR 1282 ISP Infectiologie et Santé Publique, Centre de recherche Val de Loire, Nouzilly, France
9. University of Veterinary and Animal Sciences, Lahore, 54600, Pakistan

Table of Contents

[Section 1. Chromosome structure of Haemonchus contortus 2](#_Toc52467866)

[Supplementary Figure 1. Genome assembly pipeline and improvement timeline. 2](#_Toc52467867)

[Supplementary Figure 2. Assembly of the X chromosome. 3](#_Toc52467868)

[Supplementary Figure 3. Conservation of ortholog synteny between Haemonchus contortus and Caenorhabditis elegans. 5](#_Toc52467869)

[Supplementary Table 1. Genome assembly completeness measured by detection of universally conserved orthologs using BUSCO and CEGMA 6](#_Toc52467870)

[Supplementary Figure 4. Visualisation of genome completeness metrics using BUSCO. 7](#_Toc52467871)

[Supplementary Table 2. The density of repetitive units throughout the genome identified with RepeatMasker 8](#_Toc52467872)

[Section 2. Resolving haplotypic diversity and repeat distribution within the chromosomes 9](#_Toc52467873)

[Supplementary Figure 5. Distribution of repetitive units throughout the genome identified with RepeatMasker. 9](#_Toc52467874)

[Section 3: Generation of a high-quality transcriptome annotation incorporating short and long reads 10](#_Toc52467875)

[Supplementary Figure 6. Annotation pipeline schematic used to incorporate RNA-seq and Iso-Seq data into a single annotation. 10](#_Toc52467876)

[Supplementary Table 3. Comparison of PacBio RSII and Sequel Iso-Seq reads 11](#_Toc52467877)

[Supplementary Table 4. Sensitivity and specificity of the annotation pipeline vs curated genome annotation 12](#_Toc52467878)

[Supplementary Table 5. Transcriptome characterisation of the nuclear genome 13](#_Toc52467879)

[Supplementary Table 6. Pairwise comparison of one-to-one orthologs using OrthoFinder 14](#_Toc52467880)

[Section 4: Transcriptional dynamics throughout development and between sexes 15](#_Toc52467881)

[Supplementary Figure 7. Transcript co-expression profiles across life stages of Haemonchus contortus. 15](#_Toc52467882)

[Section 5: Transcriptional complexity is defined by extensive cis- and trans-splicing 16](#_Toc52467883)

[Supplementary Figure 8. Comparison of splice leader and splice site sequence diversity 17](#_Toc52467884)

[Supplementary Table 7. Summary of differentially spliced genes and transcripts between pairs of life stages 18](#_Toc52467885)

[Section 5: Distribution of global genetic diversity throughout the chromosomes 19](#_Toc52467886)

[Supplementary Figure 9. Principal component analysis (PCA) of mitochondrial diversity 22](#_Toc52467887)

[Supplementary Figure 10. Distinct differences between mtDNA diversity in South Africa 23](#_Toc52467888)

[Supplementary Figure 11. X chromosome coverage variation 24](#_Toc52467889)

[References 25](#_Toc52467890)

# Section 1. Chromosome [structure of Haemonchus contortus](#_qj2ejurqyw7v)


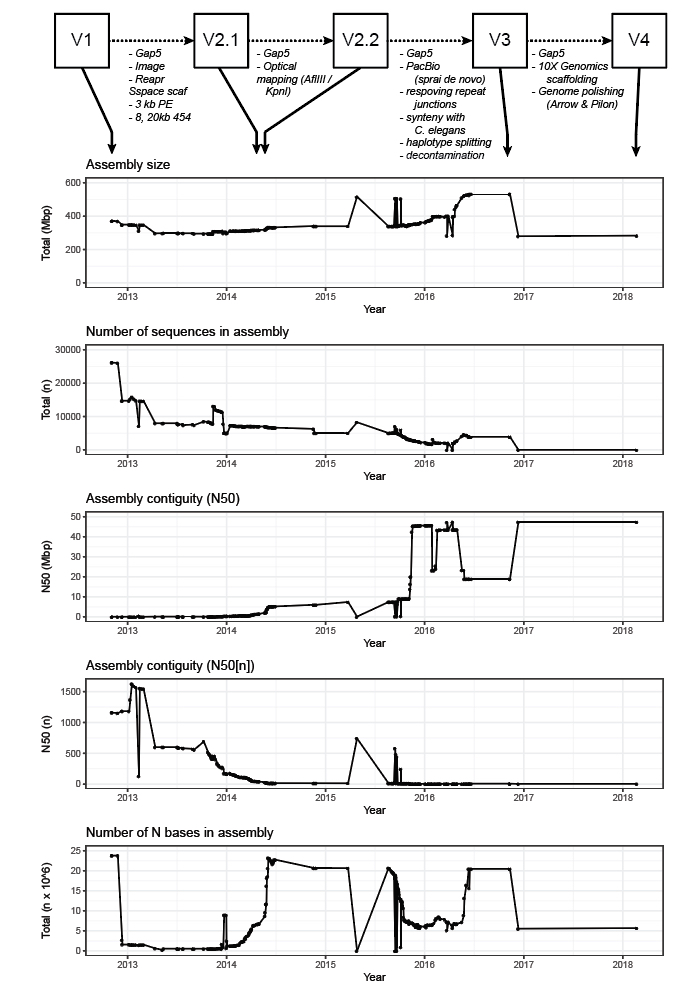


## Supplementary Figure 1. Genome assembly pipeline and improvement timeline.

The genome was iteratively improved from the published version in 2013 to the complete genome assembly presented here as new technologies became available. Here, we present the changes in the assembly over time, including the assembly size, number of contigs and/or scaffold sequences, the assembly contiguity measured using N50 and N50(n), as well as the number of N bases representing missing data. As a reference point, the V3 genome was used in the genetic map [^1^](https://paperpile.com/c/T1nTgH/UUjMi) and ivermectin backcross [^2^](https://paperpile.com/c/T1nTgH/UJbhg) analyses.


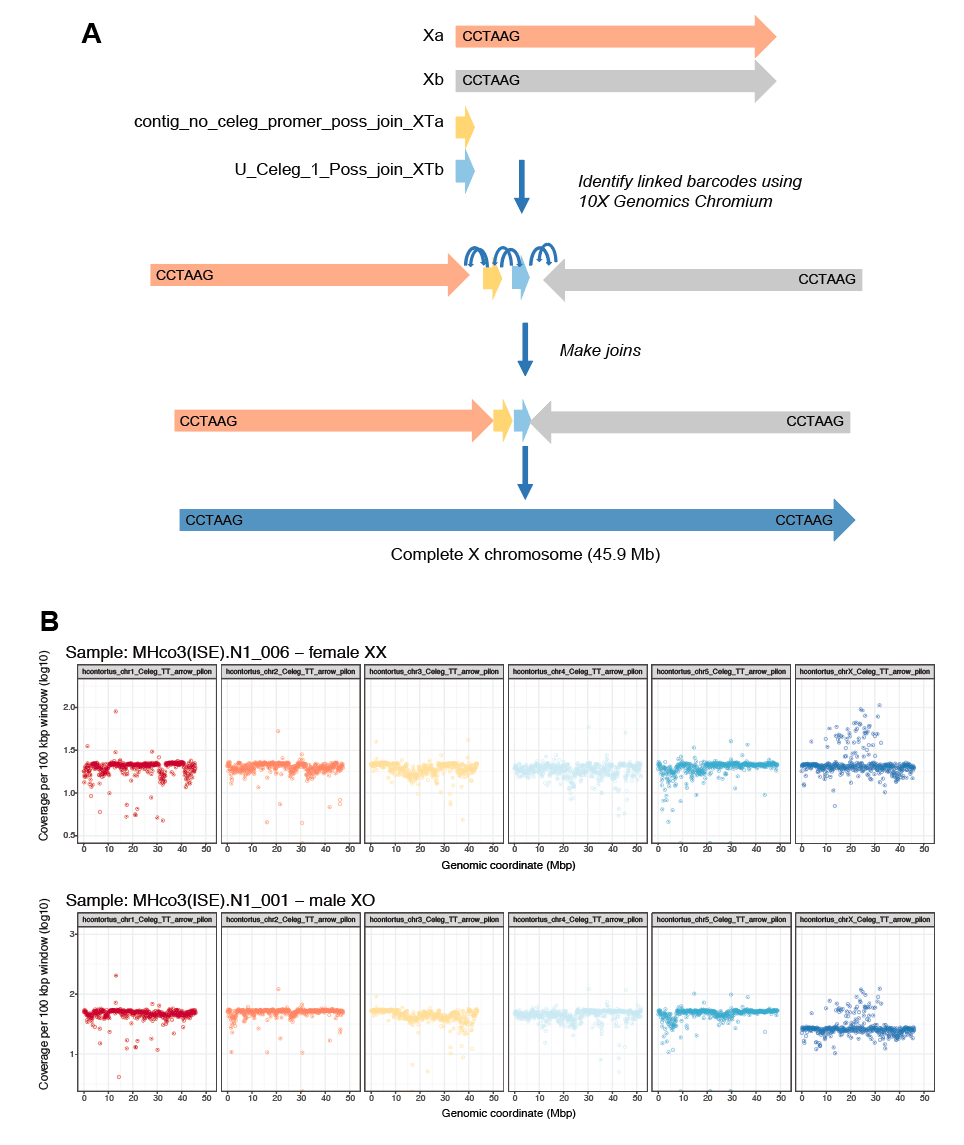


## Supplementary Figure 2. Assembly of the X chromosome.

**A.** A major update between the V3 and V4 versions of the genome was the scaffolding of the X chromosome into a single sequence. We used 10X Chromium linked reads together with the ARCS/LINKS scaffolding pipeline [^3,4^](https://paperpile.com/c/T1nTgH/v6XEs+mwjo7) to join two major scaffolds containing telomeric sequences and two short unplaced X-linked scaffolds (putatively linked to X by shared repeats and coverage differences between male and female single worm sequencing), resulting in a single 45.9 Mb X chromosome sequence. **B.** Genome-wide sequence coverage between a single XX female (top) and single XO male (bottom) parasite, illustrating the relative coverage difference of the hemizygous male X chromosome.


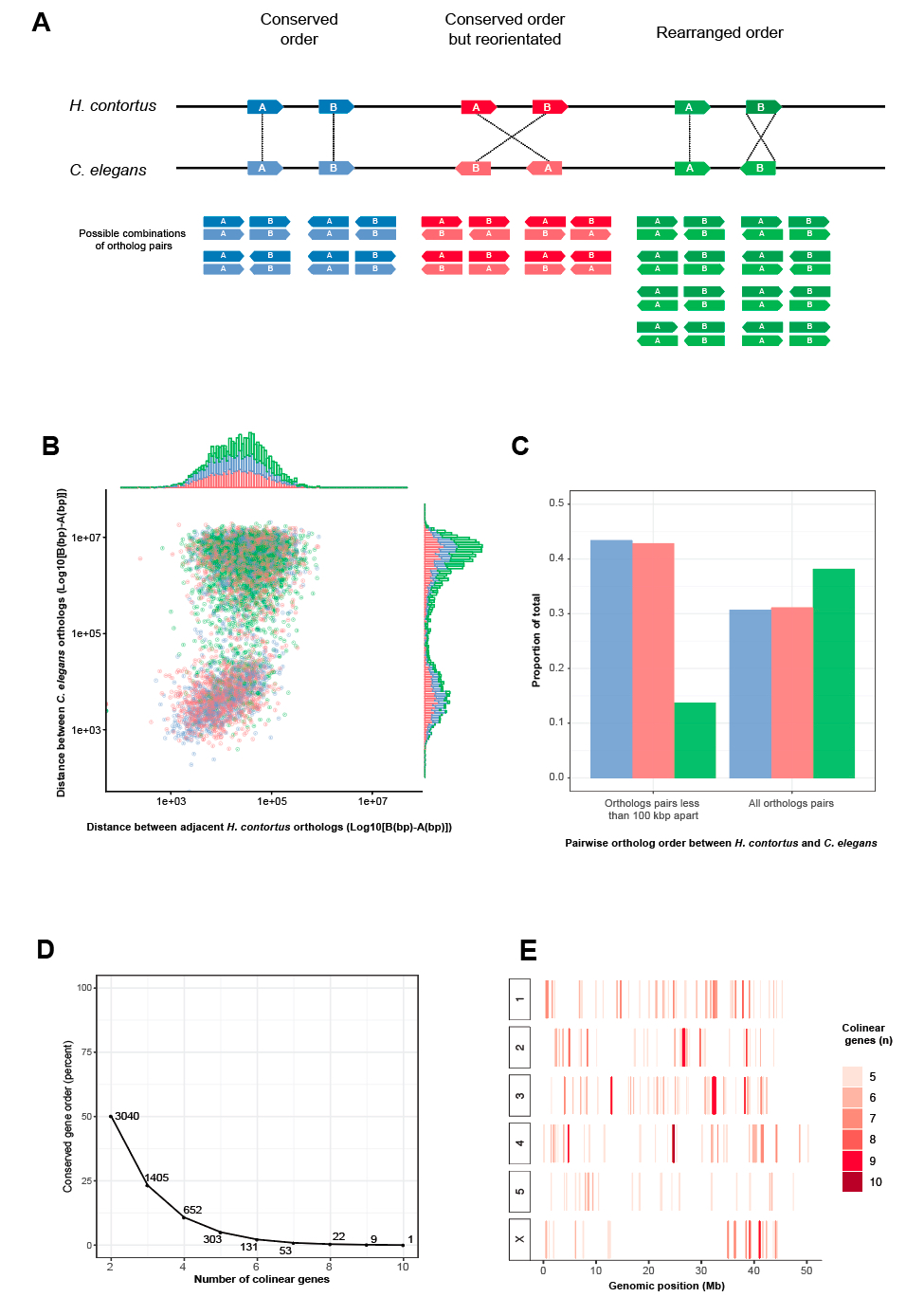


##

## Supplementary Figure 3. Conservation of ortholog synteny between Haemonchus contortus and Caenorhabditis elegans.

**A.** Schematic of the arrangement of ortholog pairs analysed. Each comparison throughout compares adjacent genes in *H. contortus* for which one-to-one orthologs were found in *C. elegans*. The experiment, therefore, examines the proportion of *H. contortus* gene pairs that have the same (or different) orientation in *C. elegans*. “Conserved order” (blue) represents pairs of orthologs that are in the same relative position and orientation between the two species. “Conserved order but reorientated” (red) represents the pairs that are in the same order, but are in a different orientation between the two species, for example, there has been a large-scale structural inversion in one species (but not the other) that changes the orientation of both genes in that species due to the inversion. “Rearranged” (green) represents instances where a change in the orientation of one of the two genes in the pair has occurred in one of the species. **B.** Scatterplot with density histograms per axis comparing the distance between adjacent *H. contortus* gene pairs (x-axis), and the relative distance between the orthologs of those gene pairs in C. elegans (y-axis). Points and bars are coloured as described in **A. C.** The proportion of pairwise groups described in **A**, comparing the frequency of those genes that were found within 100 kb of each other with all gene pairs regardless of their distance. **D.** Comparison of the proportion of colinear orthologs between the two species. Groups of colinear genes, ranging from 2 to 10 genes per group, are shown on the x-axis, and the proportion of genes in that group are shown on the y-axis. The total number of genes within each group are indicated on the plot. **E.** Genome-wide distribution of colinear blocks of orthologs between the two species, highlighting blocks with 5 or more genes.

## Supplementary Table 1. Genome assembly completeness measured by detection of universally conserved orthologs using BUSCO and CEGMA

|  | ***H. contortus V4 Chromosomes*** | ***H. contortus V4 Haplotypes*** | ***H. contortus***  ***V1*** | ***H. contortus McMaster*** | ***H. contortus New Zealand*** | ***C. elegans***  ***WB*** |
| --- | --- | --- | --- | --- | --- | --- |
| **BUSCO^1^**  **(n = 982)** |  |  |  |  |  |  |
| Complete | 859  87.4% | 574  58.4% | 774  78.8% | 613  62.5% | 853  86.8% | 969  98.7% |
| Complete +  Single copy | 843  85.8% | 493  50.2% | 601  61.2% | 584  59.5% | 781  79.5% | 963  98.1% |
| Complete +  Duplicated | 16  1.6% | 81  8.2% | 173  17.6% | 29  3.0% | 72  7.3% | 6  0.6% |
| Fragmented | 63  6.4% | 60  6.1% | 80  8.1% | 103  10.5% | 55  5.6% | 7  0.7% |
| Missing | 60  6.2% | 348  35.5% | 128  13.1% | 266  27.0% | 74  7.6% | 6  0.6% |
| **CEGMA^2^**  **(n = 248)** |  |  |  |  |  |  |
| Complete | 229  92.34% | 161  64.92% | 213  85.89% | 175  70.56% | 179  72.18% | 240  96.77% |
| Ave. orthologs (complete) | 1.1 | 1.39 | 1.59 | 1.43 | 1.42 | 1.09 |
| Partial | 243  97.98% | 178  71.77% | 233  93.95% | 221  89.11% | 226  91.13 | 246  99.19% |
| Ave. orthologs (partial) | 1.22 | 1.6 | 1.84 | 1.81 | 1.71 | 1.2 |

1. [BUSCO (Simão et al. 2015) version 3.0.2](https://paperpile.com/c/J99shT/GKhj)
2. CEGMA (Parra, Bradnam, and Korf 2007) v2.5


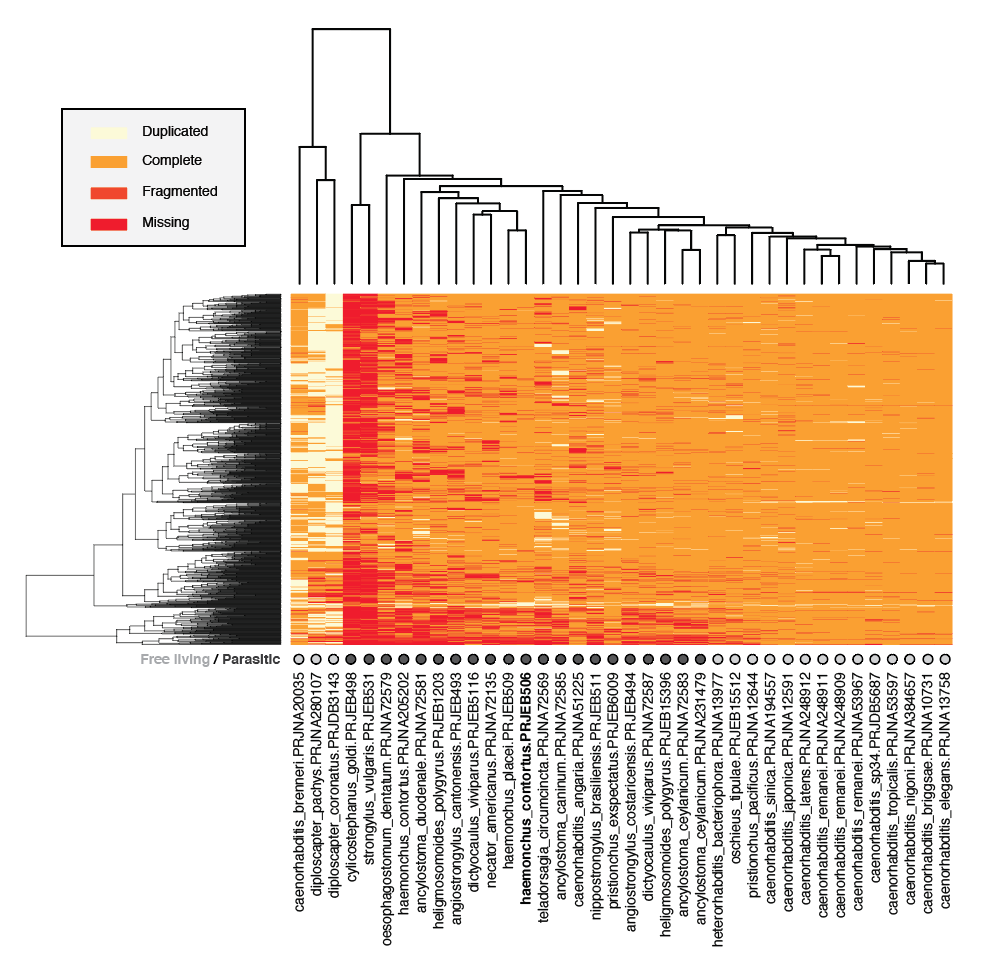


##

##

##

##

##

##

##

##

##

##

##

##

##

##

##

## Supplementary Figure 4. Visualisation of genome completeness metrics using BUSCO.

Comparison of BUSCO completeness among Clade V nematodes. Heatmap presents the complete, fragmented, missing and duplicated BUSCOs of the 982 total BUSCOs (rows) analysed in each genome assembly (columns) of the Clade V nematodes in WBP11. The *H. contortus* V4 genome is indicated in bold. Although BUSCO is used as a measure of genome completeness, we argue that some evidence of phylogenetic structure is present in the clustering of the BUSCO profiles of each species, suggesting systematic missingness due to gene gain and/or loss over evolutionary history. There is also a clear distinction that parasitic species (dark grey circles) have generally poorer BUSCO scores than free-living species (light grey circles). Note this is somewhat confounded by the fact that most free-living species are *Caenorhabditis spp.*, of which *C. elegans* was one species used to develop the BUSCO gene set.

## Supplementary Table 2. The density of repetitive units throughout the genome identified with RepeatMasker

|  | ***H. contortus V4 Chromosomes*** | | | ***H. contortus V4 Haplotypes*** | | | ***H. contortus V1*** | | |
| --- | --- | --- | --- | --- | --- | --- | --- | --- | --- |
| **Repeat type** | **Copies** | **Total bp** | **Total %** | **Copies** | **Total bp** | **Total %** | **Copies** | **Total bp** | **Total %** |
| Total sequence length |  | 283439308 |  |  | 248771548 |  |  | 369846877 |  |
| Total masked length |  | 103265398 | 36.43 |  | 78159564 | 31.42 |  | 112220283 | 30.34 |
| Total interspersed repeats | 292099 | 98394895 | 34.71 | 236979 | 74168974 | 29.81 | 376596 | 109698001 | 29.66 |
| *SINE* | 11716 | 2066138 | 0.73 | 16285 | 3170882 | 1.27 | 1332 | 113378 | 0.03 |
| *LINE* | 39451 | 18245043 | 6.44 | 26815 | 11816099 | 4.75 | 54566 | 19976741 | 5.4 |
| *LTR* | 11378 | 7345716 | 2.59 | 7273 | 4596616 | 1.85 | 9218 | 5354650 | 1.45 |
| *DNA* | 35396 | 18332560 | 6.47 | 30451 | 14756722 | 5.93 | 45885 | 21430732 | 5.79 |
| *Unclassified* | 194158 | 52405438 | 18.49 | 156155 | 39828655 | 16.01 | 265595 | 62822500 | 16.99 |
| Small RNA | 10411 | 1948687 | 0.69 | 14784 | 3082890 | 1.24 | 557 | 54712 | 0.01 |
| Satellites | 357 | 78425 | 0.03 | 25 | 73937 | 0.03 | 1100 | 214313 | 0.06 |
| Simple repeats | 30473 | 4730196 | 1.67 | 27047 | 3776806 | 1.52 | 41715 | 2359773 | 0.64 |
| Low complexity sequence | 4557 | 227074 | 0.08 | 4442 | 220326 | 0.09 | 6274 | 302055 | 0.08 |

# Section 2. Resolving haplotypic diversity and repeat distribution within the chromosomes


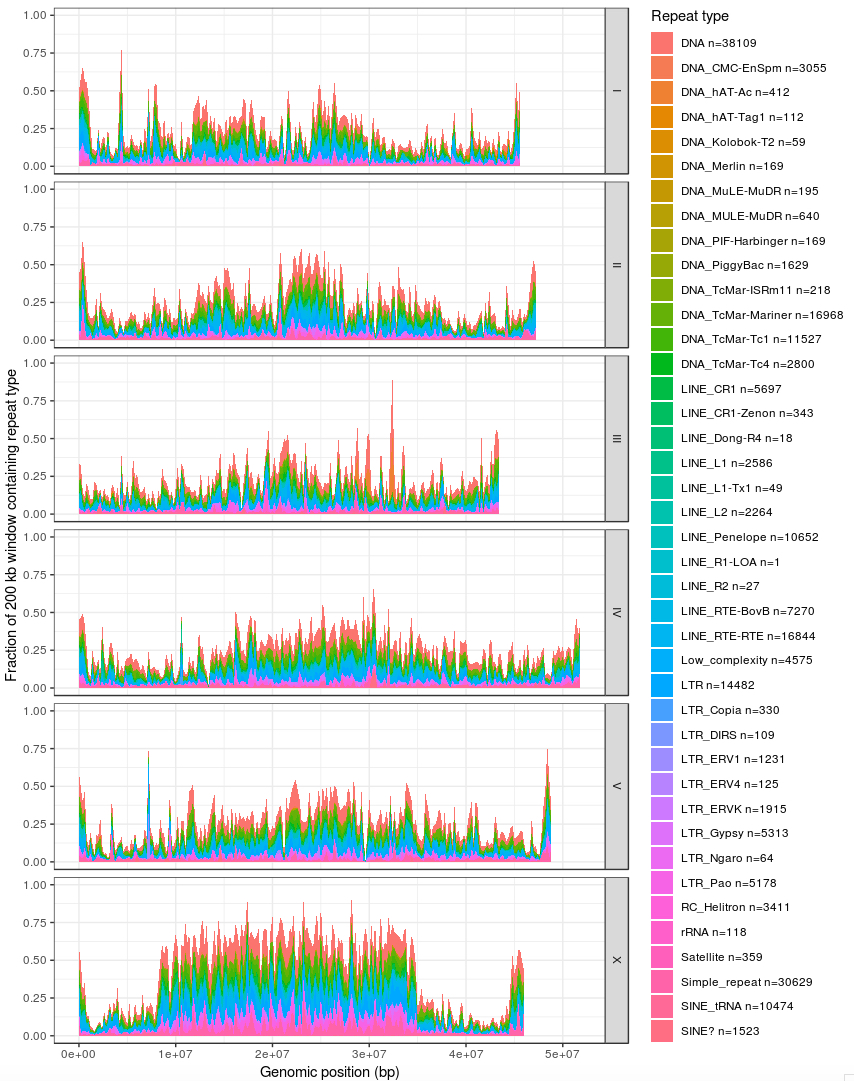


##

## Supplementary Figure 5. Distribution of repetitive units throughout the genome identified with RepeatMasker.

##

# Section 3: Generation of a high-quality transcriptome annotation incorporating short and long reads


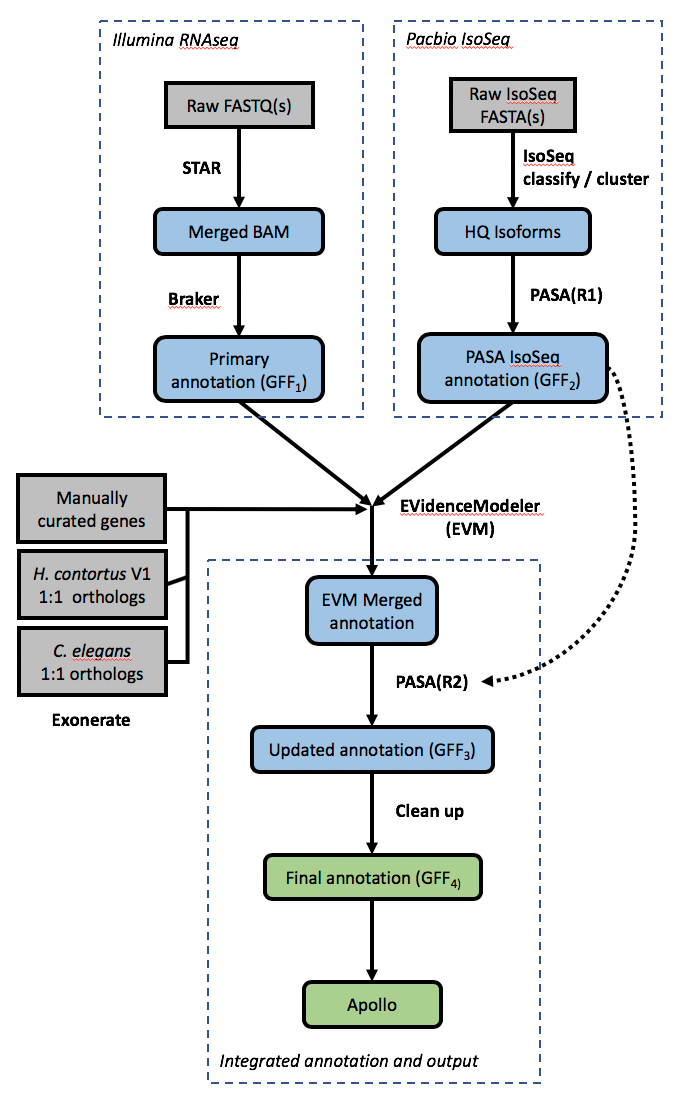


## Supplementary Figure 6. Annotation pipeline schematic used to incorporate RNA-seq and Iso-Seq data into a single annotation.

## Supplementary Table 3. Comparison of PacBio RSII and Sequel Iso-Seq reads

|  | **RSII (x5)** | **% total RSII reads** | **Sequel (X1)** | **% total Sequel reads** |
| --- | --- | --- | --- | --- |
| Reads of insert | 430,599 |  | 679,422 |  |
| Five prime reads | 314,413 | 73.02 | 378,120 | 55.65 |
| Three prime reads | 292,292 | 67.88 | 340,568 | 50.13 |
| Poly-A reads | 162,785 | 37.8 | 188,663 | 27.77 |
| Filtered short reads | 81,271 | 18.87 | 252 | 0.04 |
| Non-full-length reads | 238,135 | 55.3 | 534,496 | 78.67 |
| Full-length reads | 111,193 | 25.82 | 144,674 | 21.29 |
| Full-length non-chimeric reads | 109,511 | 25.43 | 142,018 | 20.9 |
| Full-length non-chimeric read length | 1,773 |  | 2,394 |  |

## Supplementary Table 4. Sensitivity and specificity of the annotation pipeline vs curated genome annotation

| **Reference** | **V1 curated genes^1^** | **V4 annotation iterative improvement to final version** | | |  |
| --- | --- | --- | --- | --- | --- |
| **Comparison** | **V1 annotation** | **Braker** | **PASA** | **EVM + PASA (round 2)** |  |
| *Sensitivity* |  |  |  |  |  |
| Base | 88.2 | 85.4 | 85 | 92.9 |  |
| Exon | 82.4 | 89.6 | 89.6 | 94.7 |  |
| Intron | 86.2 | 94.8 | 90.1 | 96.3 |  |
| Transcript level | 30.2 | 72.7 | 33.3 | 86.7 |  |
| Locus level | 30.6 | 78 | 30.3 | 87.5 |  |
| *Precision* |  |  |  |  |  |
| Base | 91.3 | 79.7 | 97.7 | 98.7 |  |
| Exon | 83.8 | 86.7 | 95.4 | 96.1 |  |
| Intron | 86.2 | 93.9 | 96.5 | 98 |  |
| Transcript level | 30.3 | 56.3 | 30.8 | 86.6 |  |
| Locus level | 35.1 | 60.6 | 29.8 | 86.4 |  |
|  |  |  |  |  |  |
| **BUSCO**  **(proteins)** | **V1** | **Braker** | **PASA** | **EVM +**  **PASA (round 2)** | **Final** |
| Complete | 82.90% | 86.50% | 74.90% | 87.30% | 88.40% |
| Complete + Single copy | 54.60% | 78.80% | 64.60% | 80.30% | 80.60% |
| Complete + Duplicated | 28.30% | 7.70% | 10.30% | 7% | 7.80% |
| Fragmented | 4% | 4.80% | 6% | 3.20% | 2.60% |
| Missing | 13.10% | 9.70% | 19.10% | 9.50% | 9% |

- - - 1. 599 curated genes (ABC transporters / LGiC) in the V1 genome

## Supplementary Table 5. Transcriptome characterisation of the nuclear genome

|  | ***H. contortus* V4 chromosomes** | ***H. contortus V1*** | ***H. contortus***  ***McMaster*** | ***C. elegans WB*** |
| --- | --- | --- | --- | --- |
| Protein coding genes (n) | 19489 | 23489 | 23610 | 20240 |
| mRNAs (n) | 20987 | 26367 | 24942 | 33313 |
| Exons (n) | 187017 | 243667 | 203819 | 163683 |
| Introns (n) | 166030 | 217300 | 178877 | 139847 |
| CDSs (n) | 20986 | 24747 | 24942 | 23115 |
| UTRs (n) | 23687 | 21622 | 40550 | 43219 |
| Overlapping genes (n) | 3033 | 891 | 2357 | 3893 |
| Contained genes (n) | 1643 | 461 | 509 | 631 |
| Longest gene (bp) | 447147 | 91953 | 196066 | 102626 |
| Longest mRNA (bp) | 447147 | 91953 | 196066 | 102626 |
| Mean gene length (bp) | 7677 | 5456 | 6377 | 3132 |
| Mean mRNA length (bp) | 8442 | 6031 | 6721 | 3989 |
| Mean exon length (bp) | 162 | 124 | 140 | 242 |
| Mean intron length (bp) | 887 | 594 | 780 | 419 |
| Mean CDS length (bp) | 1237 | 1218 | 909 | 1297 |
| Mean UTR length (bp) | 564 | 111 | 143 | 133 |
| Mean exons per mRNA (n) | 9 | 9 | 8 | 7 |
| Mean introns per mRNA (n) | 8 | 8 | 7 | 5 |
| Genome covered by genes (%) | 52.8 | 34.7 | 47.1 | 63.2 |
| Genome covered by CDSs (%) | 9.2 | 8.1 | 7.1 | 29.9 |

Summary statistics were generated from a GFF using the tool Genome Annotation Generator (GAG) (Geib et al. 2018).

## Supplementary Table 6. Pairwise comparison of one-to-one orthologs using OrthoFinder

|  | ***C. elegans*** | ***H. contortus***  **McMaster** | ***H. contortus* V1** | ***H. contortus***  **V4 chromosomes** | ***H. placei*** |
| --- | --- | --- | --- | --- | --- |
| *C. elegans* |  | 4424 | 4529 | 7361 | 6371 |
| *H. contortus* McMaster | 4424 |  | 6559 | 7581 | 7861 |
| *H. contortus* V1 | 4529 | 6559 |  | 9595 | 7991 |
| *H. contortus* V4 *chromosomes* | 7361 | 7581 | 9595 |  | 9970 |
| *H. placei* | 6371 | 7861 | 7991 | 9970 |  |

# Section 4: Transcriptional dynamics throughout development and between sexes

##


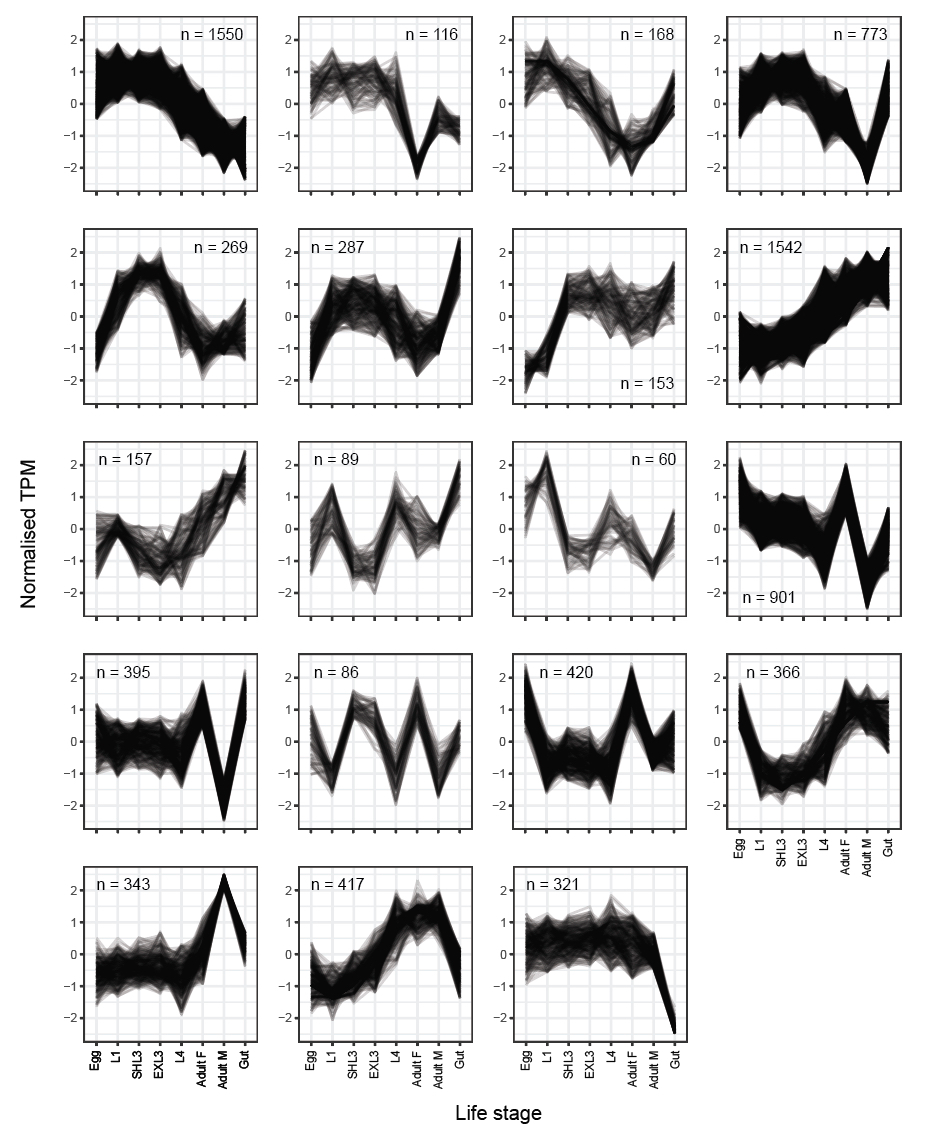


## Supplementary Figure 7. Transcript co-expression profiles across life stages of Haemonchus contortus.

Transcript abundance, defined here as Transcripts per Million (TPM), was determined using Kallisto [^5^](https://paperpile.com/c/T1nTgH/P18Hg). Automated clustering of 20,986 transcripts using *clust* [^6^](https://paperpile.com/c/T1nTgH/1MkiT) identified 19 clusters comprising a total of 8412 transcripts with shared differential expression across the life stages, indicative of putatively co-regulated transcripts.

# Section 5: Transcriptional complexity is defined by extensive cis- and trans-splicing


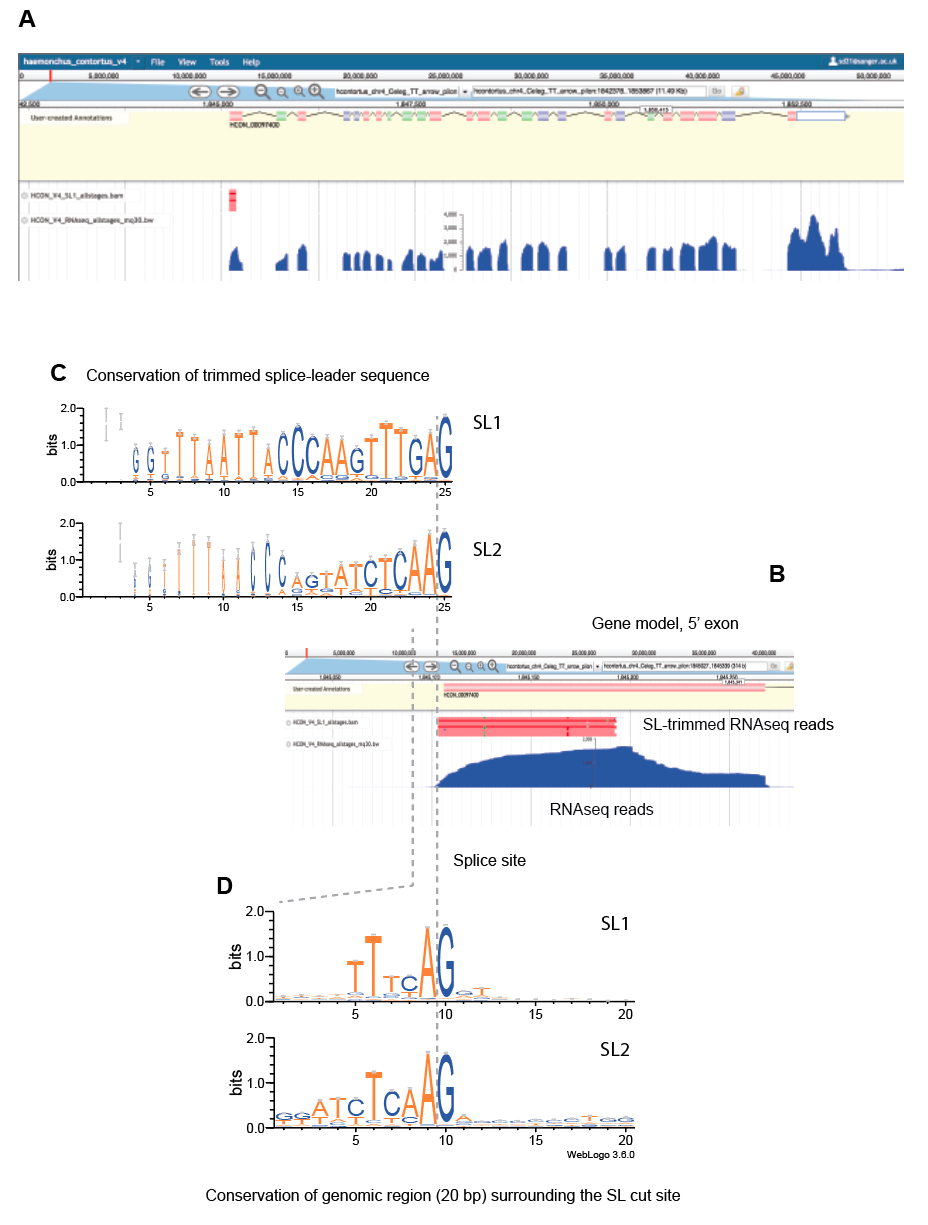


## Supplementary Figure 8. Comparison of splice leader and splice site sequence diversity

**A.** Apollo screenshot of the HCON_00097400 gene model, SL1-trimmed RNA-seq reads (red trimmed reads), and RNA-seq data (blue bigwig plot) supporting the gene model. **B.** Zoomed view of the 5’ end of the gene model, highlighting the hard-trimmed RNA-seq reads indicative of a match and removal of the SL1 sequence at the same genomic coordinates. This represents the splice site. **C.** Conservation of the DNA sequence containing the trimmed SL1 or SL2 sequence, visualised using WebLogo. The decrease in sequence conservation upstream and away from the splice site is largely due to the decrease in RNA-seq read coverage as a result of mRNA decay. **D.** Conservation of genomic DNA sequence surrounding the splice site coordinates, demonstrating the high conservation of AG splice site, but also, enrichment in sequence motifs that differ between SL1- and SL2-targeted genes.

#

## Supplementary Table 7. Summary of differentially spliced genes and transcripts between pairs of life stages

| **Life stage transition** | **Genes passing**  **filtering** | **Genes with one or more introns differentially spliced (FDR < 0.05)** | **Introns passing filter** | **Introns with differential splicing (FDR < 0.05)** |
| --- | --- | --- | --- | --- |
| Egg > L1 | 974 | 273 (28.03) | 1337 | 335 (25.0) |
| L1 > SHL3 | 1156 | 418 (36.16) | 1537 | 494 (32.1) |
| SHL3 > EXL3 | 1108 | 43 (3.88) | 1455 | 43 (2.9) |
| EXL3 > L4 | 982 | 417 (42.46) | 1293 | 510 (39.4) |
| L4 > Adult Male | 1144 | 342 (29.90) | 1546 | 406 (26.2) |
| L4 > Adult Female | 934 | 296 (31.69) | 1204 | 343 (28.4) |
| Adult Male > Adult Female | 1068 | 231 (21.63) | 1404 | 247 (17.5) |
| Adult Female > Gut | 759 | 204 (26.88) | 958 | 216 (22.5) |
| Adult Female > Egg | 931 | 324 (34.80) | 1237 | 406 (32.8) |

#

# Section 5: Distribution of global genetic diversity throughout the chromosomes


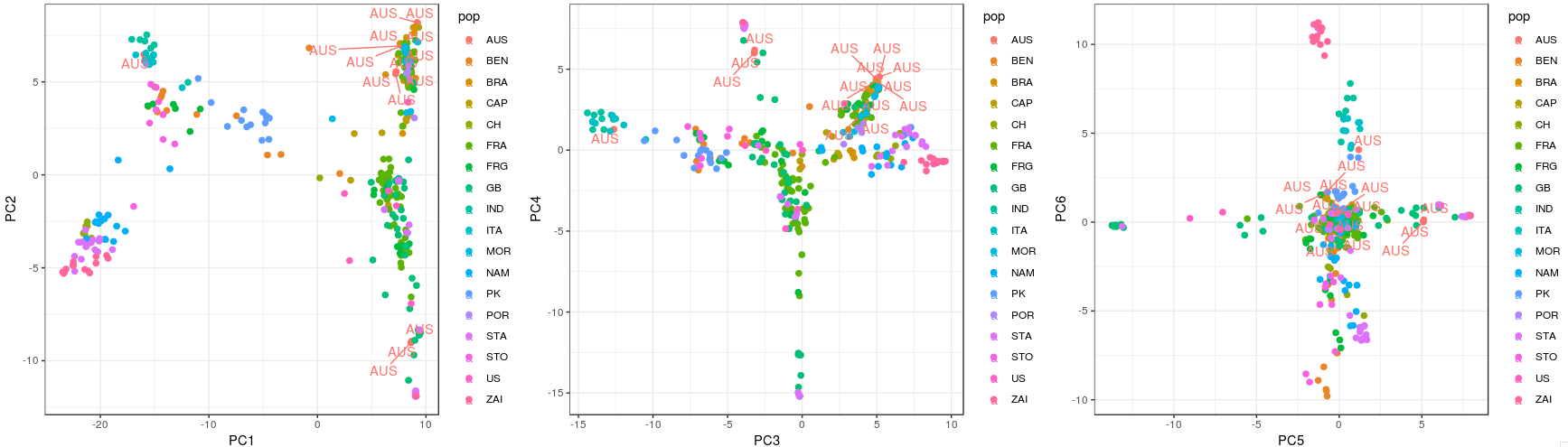


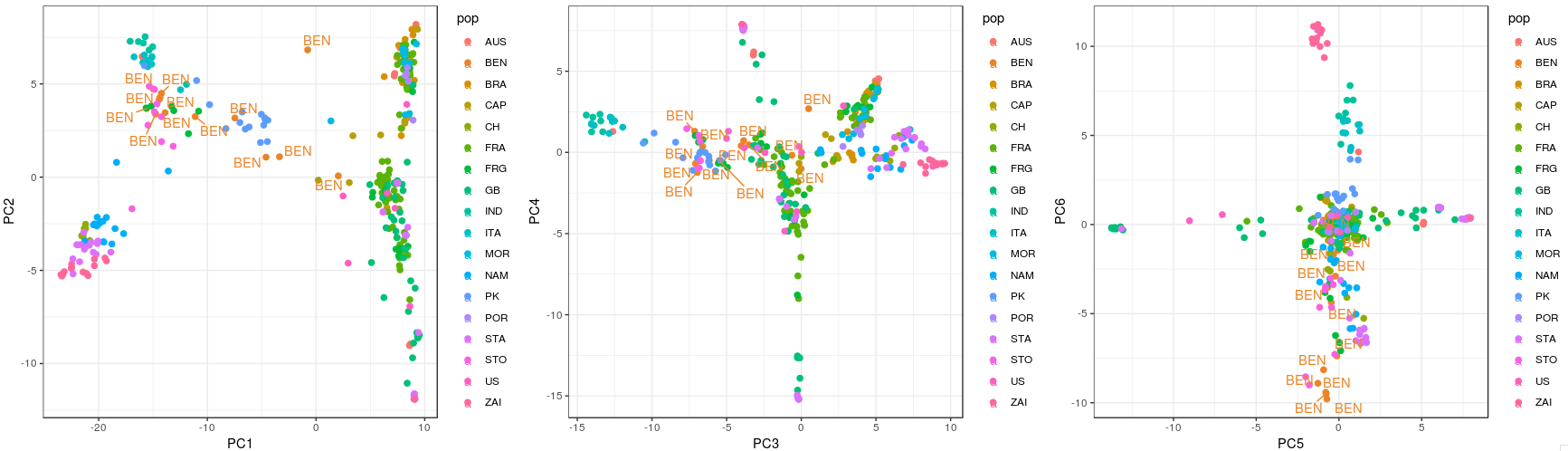


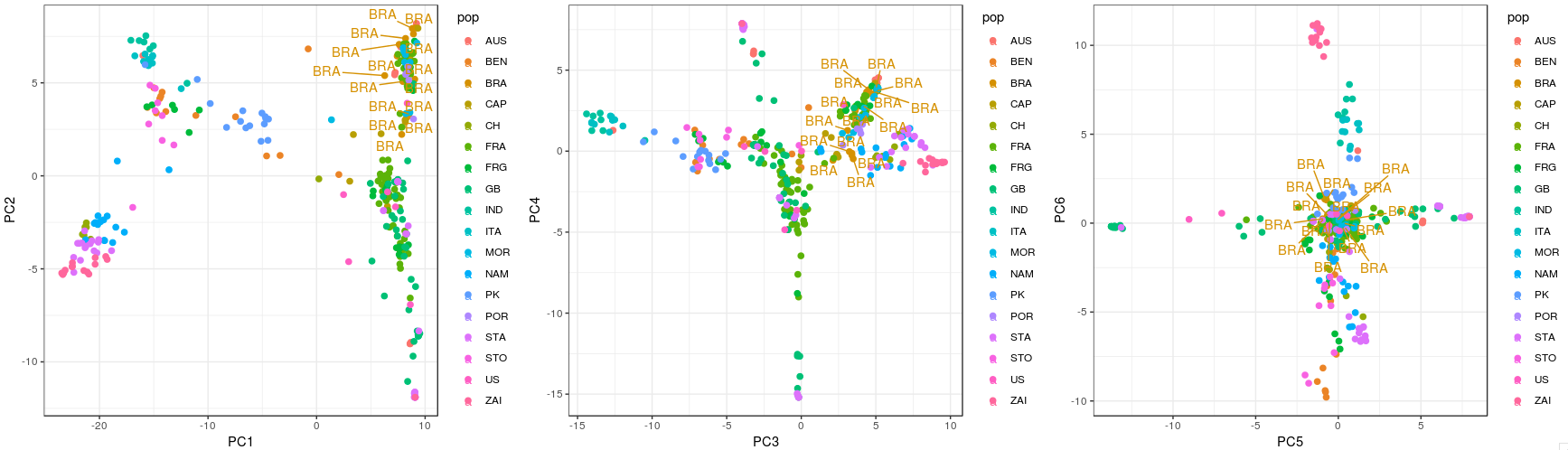


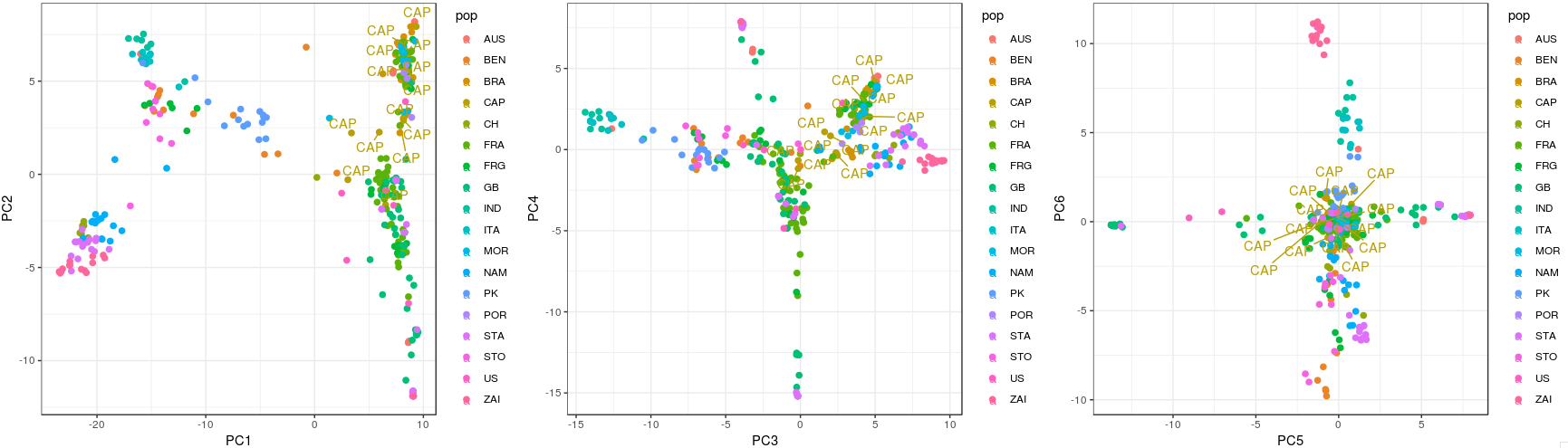


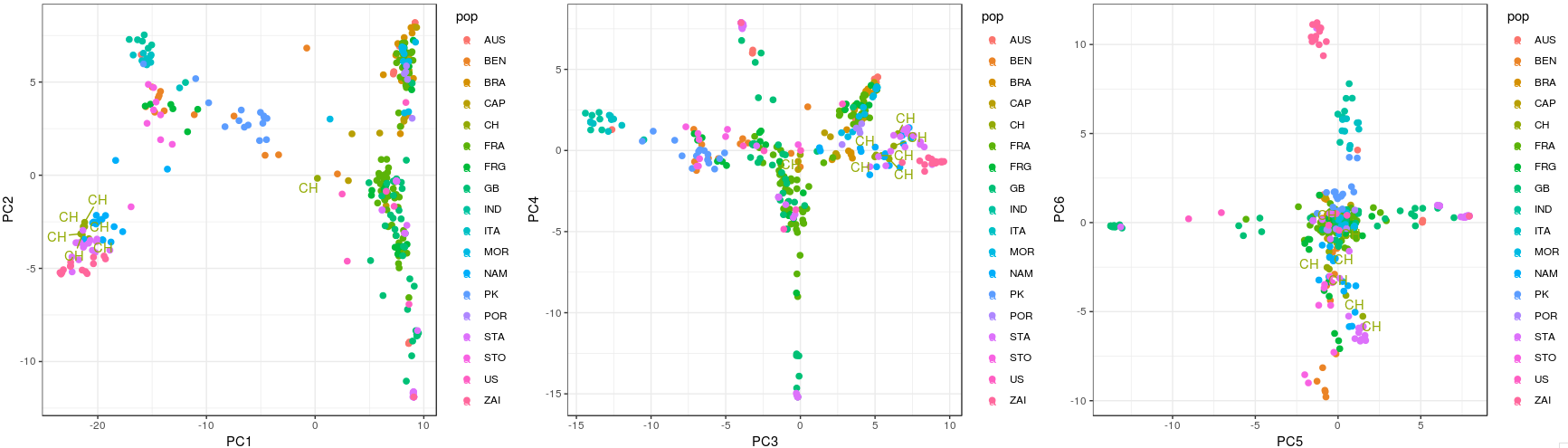

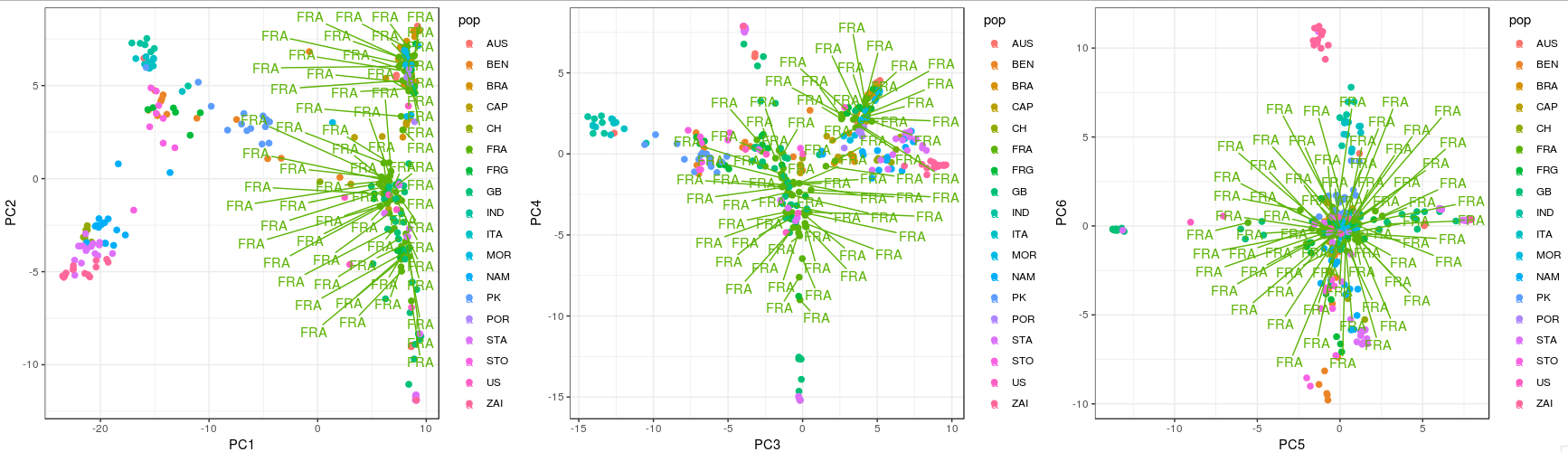


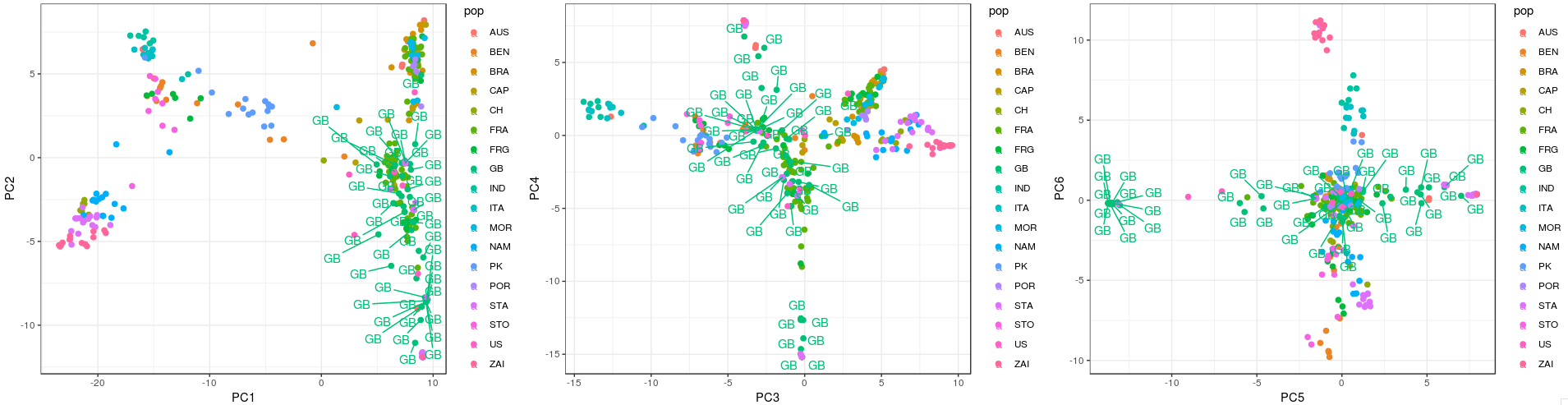


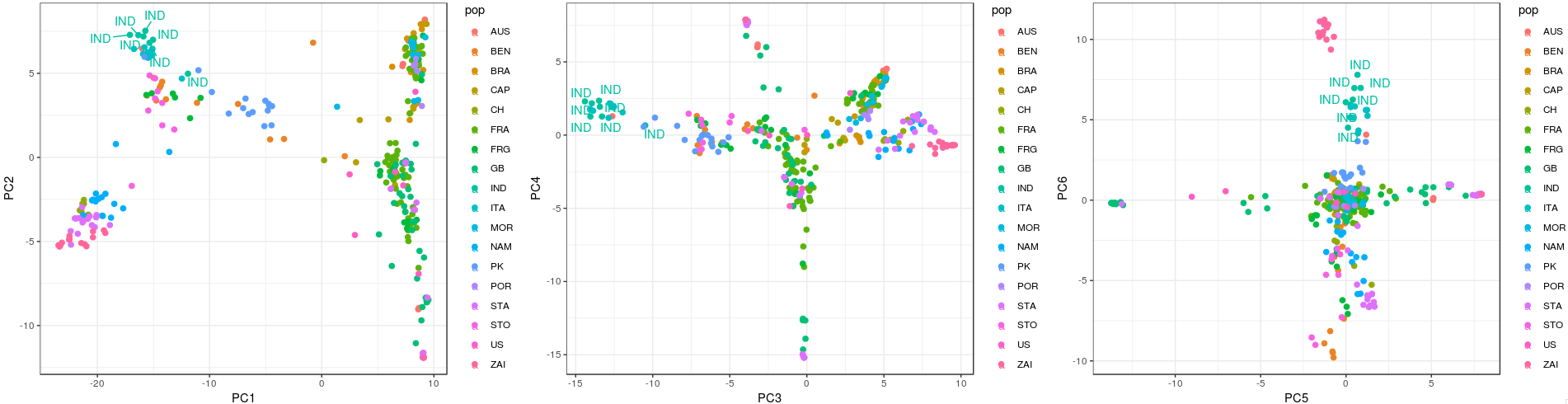


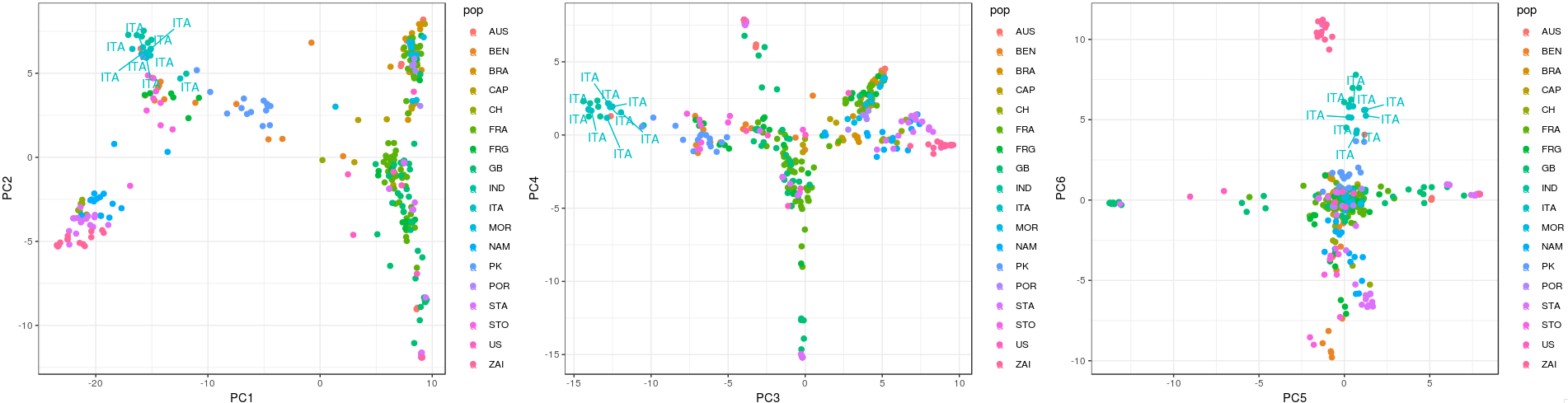


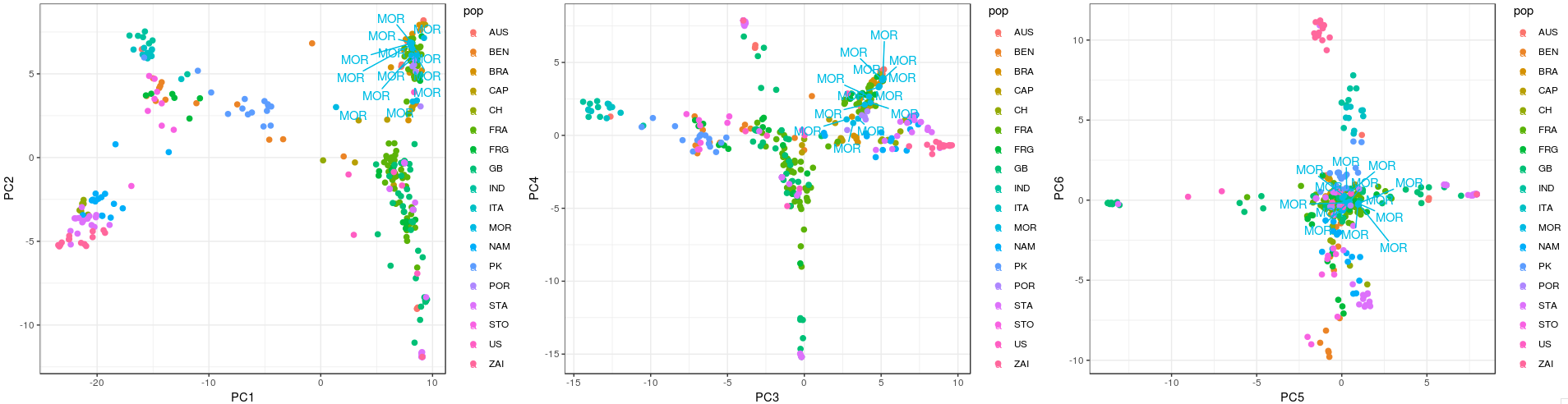


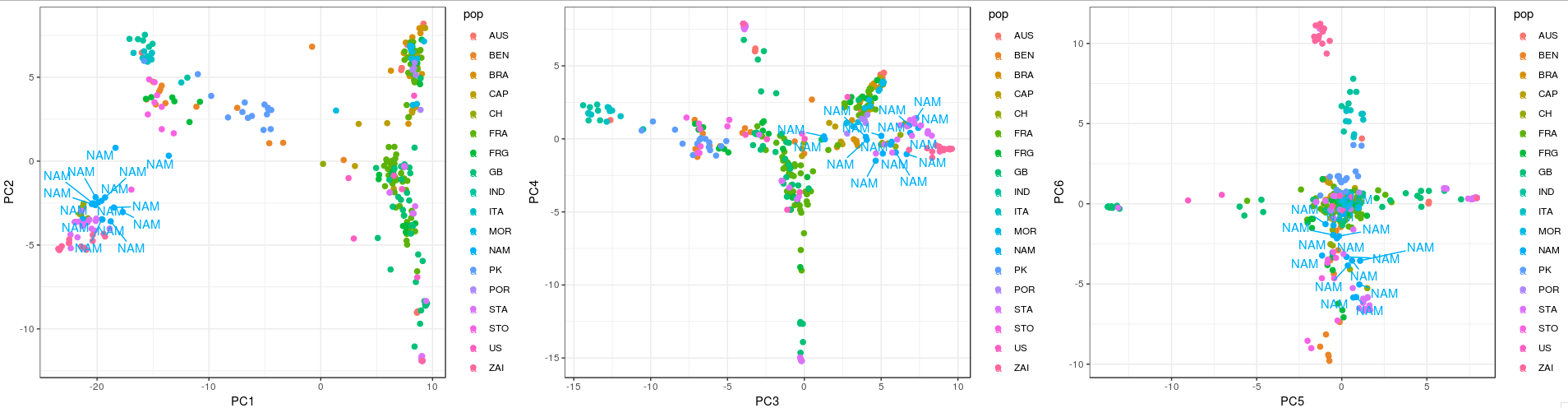


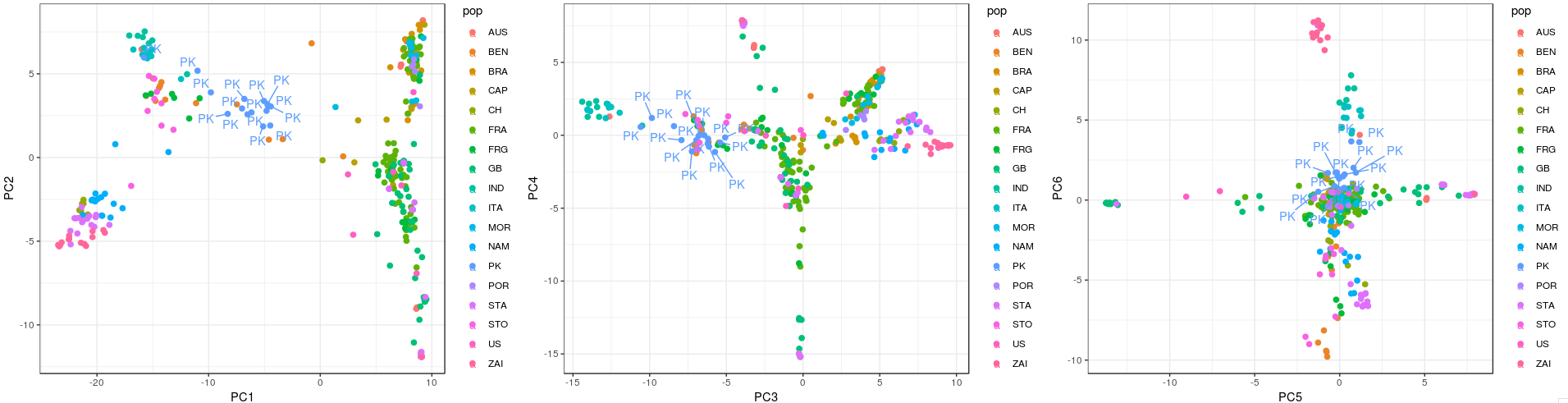


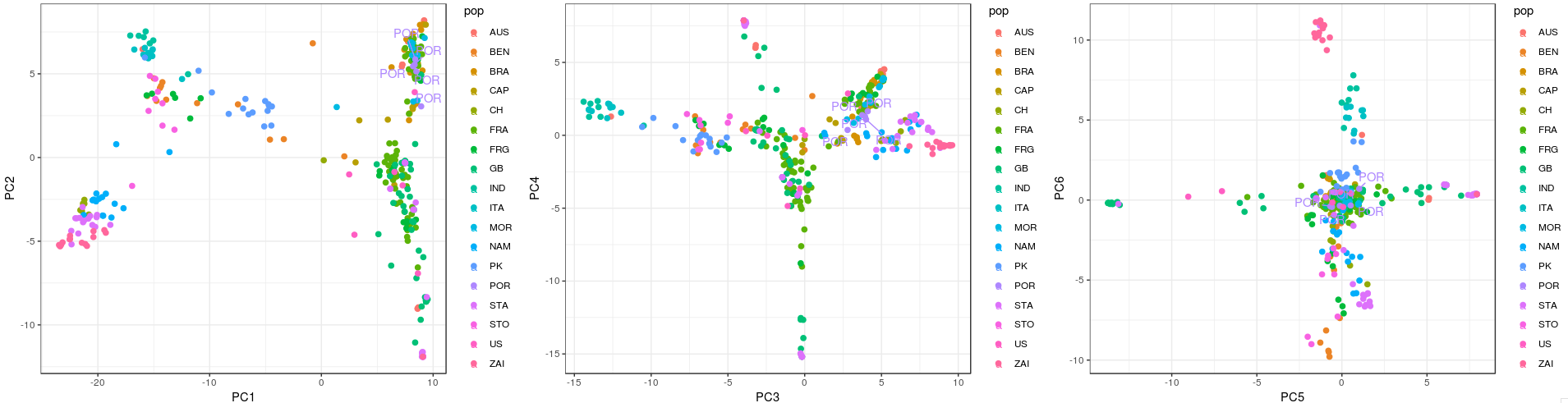


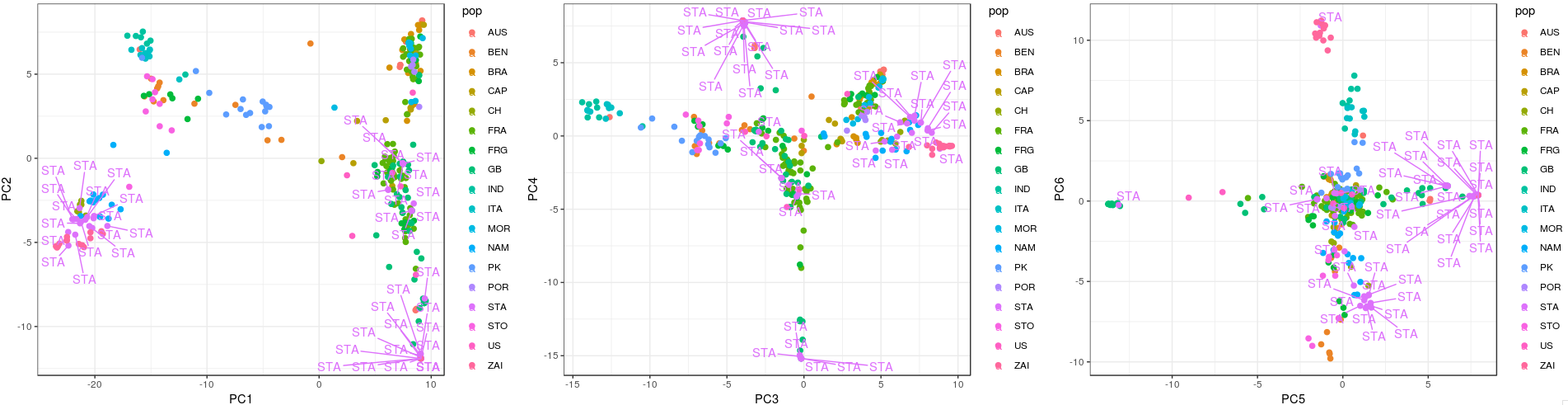


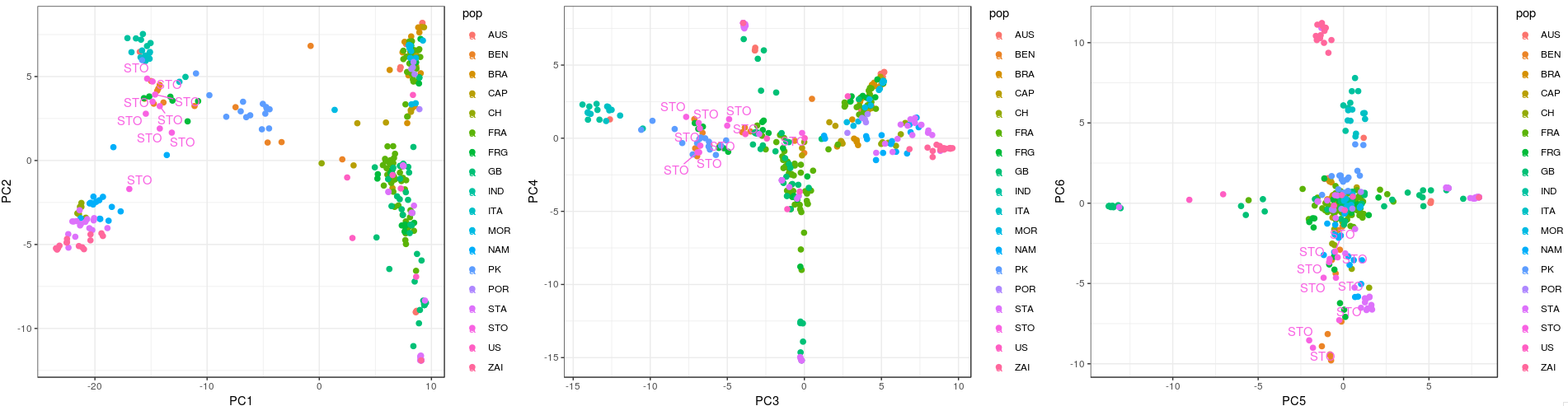


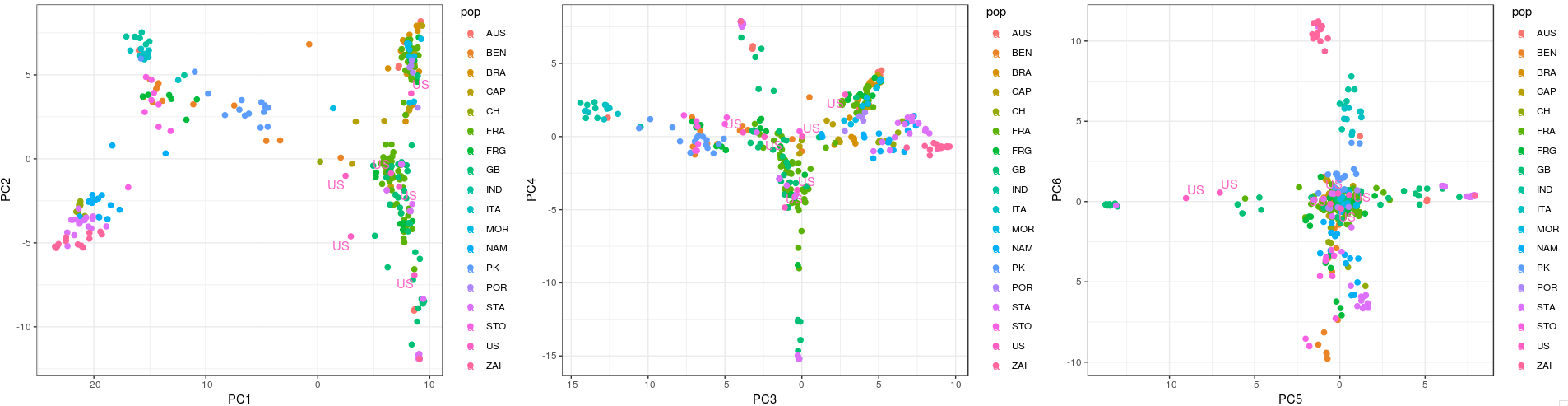


## Supplementary Figure 9. Principal component analysis (PCA) of mitochondrial diversity

We extend the analysis of genetic diversity shown in Figure 6 B of the main text to show additional principal component comparisons (PC1v2, PC3v4, PC5v6) that are labelled to highlight clustering of samples by country. Countries: Australia (AUS), Benin (BEN), Brazil (BRA), Cape Verde (CAP), Switzerland (CH), France (FRA), Guadeloupe (FRG), United Kingdom (GB), Indonesia (IND), Italy (ITA), Morocco (MOR), Namibia (NAM), Pakistan (PK), Portugal (POR), South Africa (STA), Sao Tome (STO), and United States (US).

##

##

##

##

##

##


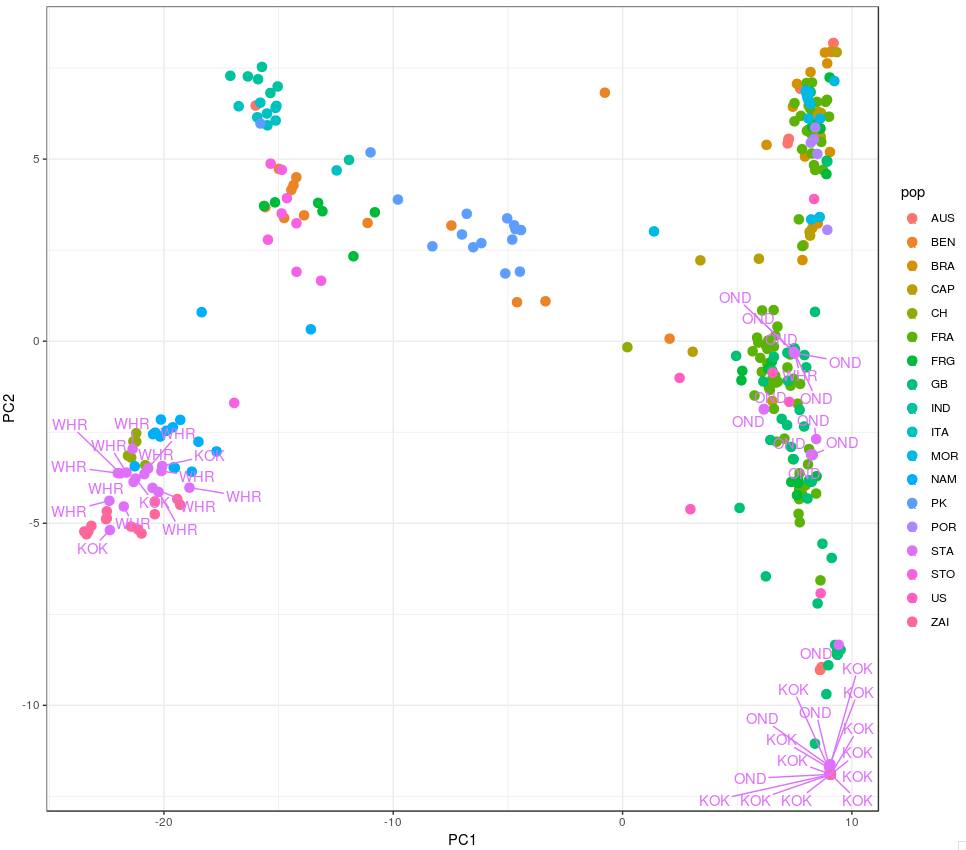


##

## Supplementary Figure 10. Distinct differences between mtDNA diversity in South Africa

Three South African populations of parasites are included, White River (WHR), Kokstadt (KOK), and Onderstepoort (OND). Despite their proximity geographically, they are genetically distinct.

##

##

##

##


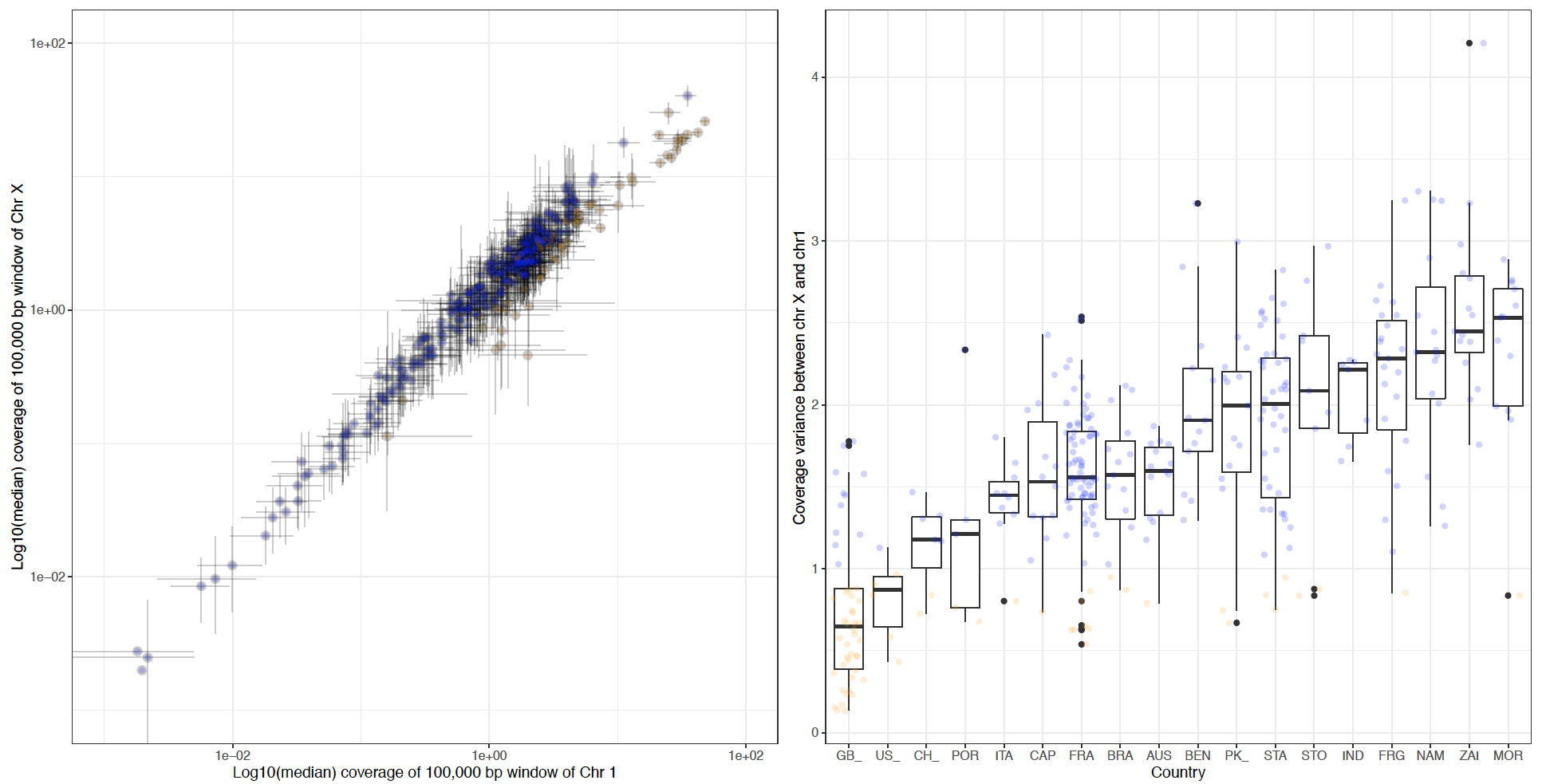


##

## Supplementary Figure 11. X chromosome coverage variation

**A.** Coverage variation between the X chromosome and chromosome I per sample. Error bars represent the 1st (min) and 3rd (max) quartiles of the coverage distribution for the X (y-axis) and autosome (x-axis). **B.** Coverage variation of the X chromosome, normalised by the coverage on the autosomes, per population. Populations are ordered by median coverage from lowest median variation to highest. In both plots, points are coloured by the X-to-autosome coverage ratio greater than (blue) or less than (yellow) 1.

##

##

##

##

##

##

##

##

#

# References

1. [Doyle, S. R. *et al.* A Genome Resequencing-Based Genetic Map Reveals the Recombination Landscape of an Outbred Parasitic Nematode in the Presence of Polyploidy and Polyandry. *Genome Biol. Evol.* **10**, 396–409 (2018).](http://paperpile.com/b/T1nTgH/UUjMi)

2. [Doyle, S. R. *et al.* Population genomic and evolutionary modelling analyses reveal a single major QTL for ivermectin drug resistance in the pathogenic nematode, Haemonchus contortus. *BMC Genomics* **20**, 218 (2019).](http://paperpile.com/b/T1nTgH/UJbhg)

3. [Yeo, S., Coombe, L., Warren, R. L., Chu, J. & Birol, I. ARCS: scaffolding genome drafts with linked reads. *Bioinformatics* **34**, 725–731 (2018).](http://paperpile.com/b/T1nTgH/v6XEs)

4. [Warren, R. L. *et al.* LINKS: Scalable, alignment-free scaffolding of draft genomes with long reads. *Gigascience* **4**, 35 (2015).](http://paperpile.com/b/T1nTgH/mwjo7)

5. [Bray, N. L., Pimentel, H., Melsted, P. & Pachter, L. Near-optimal probabilistic RNA-seq quantification. *Nat. Biotechnol.* **34**, 525–527 (2016).](http://paperpile.com/b/T1nTgH/P18Hg)

6. [Abu-Jamous, B. & Kelly, S. Clust: automatic extraction of optimal co-expressed gene clusters from gene expression data. *Genome Biol.* **19**, 172 (2018).](http://paperpile.com/b/T1nTgH/1MkiT)
