# Supplementary material for: Genomic and transcriptomic variation defines the chromosome-scale assembly of Haemonchus contortus, a model gastrointestinal worm
Source: Commun Biol. 2020 Nov 9;3:656. doi: 10.1038/s42003-020-01377-3 (PMC7652881; doi:10.1038/s42003-020-01377-3)
Supplement: Supplementary file 3 — Description of Additional Supplementary Files [file 42003_2020_1377_MOESM3_ESM.docx]

**File Name:** Supplementary Data

**File Description:** Supporting data for the manuscript with the following tabs:

- Supplementary Data 1. Sequencing data used in this study.

- Supplementary Data 2. Genes within colinear blocks of shared orthologs between Haemonchus contortus and Caenorhabditis elegans

- Supplementary Data 3. Differentially expressed transcripts from pairwise comparisons of the life stages throughout the life cycle

- Supplementary Data 4. Clusters of genes sharing co-expression profiles throughout the life cycle

- Supplementary Data 5. Analysis of enriched motif sequences among co-expressed genes

- Supplementary Data 6. Caenorhabditis elegans genes and Haemonchus contortus orthologs putatively involved in dosage compensation and sex determination

- Supplementary Data 7. Lists of genes with putative SL1 and SL2 leader sequences

- Supplementary Data 8. Genes with at least one differentially spliced intron between pairs of life stages

- Supplementary Data 9. Genes within top 1% of FST outlier regions per chromosome
