# Supplementary material for: Genomic and transcriptomic variation defines the chromosome-scale assembly of Haemonchus contortus, a model gastrointestinal worm
Source: Commun Biol. 2020 Nov 9;3:656. doi: 10.1038/s42003-020-01377-3 (PMC7652881; doi:10.1038/s42003-020-01377-3)
Supplement: Supplementary file 5 — Reporting Summary [file 42003_2020_1377_MOESM5_ESM.pdf]

nature research

Corresponding author(s): Stephen R. Doyle, James A. Cotton

Last updated by author(s): Sept 15 2020

# Reporting Summary

Nature Research wishes to improve the reproducibility of the work that we publish. This form provides structure for consistency and transparency in reporting. For further information on Nature Research policies, see Authors & Referees and the Editorial Policy Checklist .

## Statistics

For all statistical analyses, confirm that the following items are present in the figure legend, table legend, main text, or Methods section.

| n/a | Confirmed | |
|---|---|---|
| ☐ | ☒ | The exact sample size (*n*) for each experimental group/condition, given as a discrete number and unit of measurement |
| ☐ | ☒ | A statement on whether measurements were taken from distinct samples or whether the same sample was measured repeatedly |
| ☐ | ☒ | The statistical test(s) used AND whether they are one- or two-sided<br>*Only common tests should be described solely by name; describe more complex techniques in the Methods section.* |
| ☐ | ☒ | A description of all covariates tested |
| ☐ | ☒ | A description of any assumptions or corrections, such as tests of normality and adjustment for multiple comparisons |
| ☐ | ☒ | A full description of the statistical parameters including central tendency (e.g. means) or other basic estimates (e.g. regression coefficient) AND variation (e.g. standard deviation) or associated estimates of uncertainty (e.g. confidence intervals) |
| ☐ | ☒ | For null hypothesis testing, the test statistic (e.g. *F*, *t*, *r*) with confidence intervals, effect sizes, degrees of freedom and *P* value noted<br>*Give P values as exact values whenever suitable.* |
| ☒ | ☐ | For Bayesian analysis, information on the choice of priors and Markov chain Monte Carlo settings |
| ☐ | ☒ | For hierarchical and complex designs, identification of the appropriate level for tests and full reporting of outcomes |
| ☒ | ☐ | Estimates of effect sizes (e.g. Cohen's *d*, Pearson's *r*), indicating how they were calculated |

*Our web collection on statistics for biologists contains articles on many of the points above.*

## Software and code

Policy information about availability of computer code

| | |
|---|---|
| Data collection | Genomic data collection is via an established production pipeline at the Wellcome Sanger Institute according to institutional policy. Sample and study management for sequencing is curated in a single laboratory management system (SequenceScape, WSI), which includes all information including sample collection, sample processing, sequencing, and quality control. |
| Data analysis | The raw sequencing data were processed and analysed using publicly available tools as described in the main text. Additional code and workflows used to analyse data are available at https://github.com/stephenrdoyle/hcontortus_genome. |

For manuscripts utilizing custom algorithms or software that are central to the research but not yet described in published literature, software must be made available to editors/reviewers. We strongly encourage code deposition in a community repository (e.g. GitHub). See the Nature Research guidelines for submitting code & software for further information.

## Data

Policy information about availability of data

All manuscripts must include a data availability statement. This statement should provide the following information, where applicable:
- Accession codes, unique identifiers, or web links for publicly available datasets
- A list of figures that have associated raw data
- A description of any restrictions on data availability

The raw sequence data is available under the ENA accession PRJEB506, with reference to specific sequencing libraries described throughout the text, and/or in Table S3 of Laing et al. 2013. RNAseq data is available from the ENA study ID PRJEB1360. The genome assembly has been made available at ENA (assembly accession: GCA_000469685.2) and WormBase ParaSite (https://parasite.wormbase.org/Haemonchus_contortus_prjeb506/Info/Index). A static version of the genome annotation used in this paper is available at ftp://ftp.sanger.ac.uk/pub/pathogens/sd21/HCON_V4_GENOME/ (signoff date: 25th Jan 2019), however, the most up-to-date version of the annotation can be accessed at WormBase ParaSite (https://parasite.wormbase.org/Haemonchus_contortus_prjeb506/Info/Index/; [15]). Genome variation data can be visualised using MicroReact (https://microreact.org/project/hcontortus_global_diversity), from which links to ENA accessions for individual sample sequencing data from the global collection can be obtained. There are no restrictions on data availability.

# Field-specific reporting

Please select the one below that is the best fit for your research. If you are not sure, read the appropriate sections before making your selection.

☐ Life sciences ☐ Behavioural & social sciences ☒ Ecological, evolutionary & environmental sciences

For a reference copy of the document with all sections, see nature.com/documents/nr-reporting-summary-flat.pdf

# Ecological, evolutionary & environmental sciences study design

All studies must disclose on these points even when the disclosure is negative.

| | |
|---|---|
| Study description | The study describes the chromosomal genome assembly, annotation, and comparitive genomic analyses of the gastrointestinal nematode pathogen Haemonchus contortus |
| Research sample | Haemonchus contortus is a gastrointestinal nematode of wild and domesticated ruminants worldwide. The samples used in this study were derived from experimentally controlled infections of the pathogen in sheep, or, from archival material (both parasite material and previously analysed sequencing data) stored after collection from the field. Sequenced samples were derived from different life-stages and from difference sexes for the parasite, as described in the manuscript |
| Sampling strategy | No statistical sampling design was applied in this study. The material was either collected specially for the genome assembly associated sequencing from controlled infections, or was obtained opportunistically from archival material. |
| Data collection | Parasitological data was primarily collected at the time of parasitological passage and sampling at the Moredun Research Institute. Experimental and field-collected sample metadata were collated at the Wellcome Sanger Institue, and made available with the manuscript. DNA and RNA data were generated at the Wellcome Sanger Institute, where samples and data are tracked using a central sample management pipeline, and is stored in the longer term at the European Nucleotide Archive (ENA) at the European Bioinformatics Institute (EBI). |
| Timing and spatial scale | Parasites samples were collected when necessary from experimentally controlled infections that were established before the draft genome assembly was published in 2013, and ongoing infections since this time. Archival samples for the global diversity dataset were collected as early as 1995 (INRA Biobank samples, sequneced and analysed in Salle et al 2019 Nature Communications), where most recent samples were sequenced in 2018. |
| Data exclusions | Data inclusion and filtering to exclude data and/or samples are described in the materials and methods of the manuscript. |
| Reproducibility | Sequencing data were generated using previosuly described protocols and analysed using publically available tools as descriebd in the text. Any additional code use for analysis and/or figure generation is described here: https://github.com/stephenrdoyle/hcontortus_genome. |
| Randomization | Randomisation was not relevant to this study. Samples were stratified by sex or life-stage, or location of origin. |
| Blinding | Blinding was not relevant for this study. The material was either collected specially for the genome sequencing from controlled infections, or was obtained opportunistically from archival material. In the latter case, it was important to know exactly where samples came from. |

Did the study involve field work? ☐ Yes ☒ No

# Reporting for specific materials, systems and methods

We require information from authors about some types of materials, experimental systems and methods used in many studies. Here, indicate whether each material, system or method listed is relevant to your study. If you are not sure if a list item applies to your research, read the appropriate section before selecting a response.

## Materials & experimental systems

| n/a | Involved in the study | |
|---|---|---|
| ☒ | ☐ | Antibodies |
| ☒ | ☐ | Eukaryotic cell lines |
| ☒ | ☐ | Palaeontology |
| ☐ | ☒ | Animals and other organisms |
| ☒ | ☐ | Human research participants |
| ☒ | ☐ | Clinical data |

## Methods

| n/a | Involved in the study | |
|---|---|---|
| ☒ | ☐ | ChIP-seq |
| ☒ | ☐ | Flow cytometry |
| ☒ | ☐ | MRI-based neuroimaging |

## Animals and other organisms

Policy information about studies involving animals; ARRIVE guidelines recommended for reporting animal research

| | |
|---|---|
| Laboratory animals | The experimental use of H. contortus requires sheep (Ovis aries) as a host organism to complete the parasites life cycle. The sheep used in this study were born and raised indoors at the Moredun Research Institute under worm free conditions. |
| Wild animals | The study did not involve wild animals. |
| Field-collected samples | Field-collected parasite samples were stored in liquid nitrogen or at -80C prior to use in the laboratory. |
| Ethics oversight | The use of experimental animals to maintain parasite populations for the purposes described in this manuscript was approved by the Moredun Research Institute Experiments and Ethics Committee and were conducted under approved British Home Office licenses in accordance with the Animals (Scientific Procedures) Act of 1986. The Home Office licence numbers were PPL 60/03899 and 60/4421, and the experimental identifiers for these studies were E06/58, E06/75, E09/36 and E14/30. |

Note that full information on the approval of the study protocol must also be provided in the manuscript.
